# Somatotopic Mapping of the Developing Sensorimotor Cortex in the Preterm Human Brain

**DOI:** 10.1093/cercor/bhy050

**Published:** 2018-04-18

**Authors:** S Dall’Orso, J Steinweg, A G Allievi, A D Edwards, E Burdet, T Arichi

**Affiliations:** 1 Department of Bioengineering, Imperial College London, London, UK; 2 Centre for the Developing Brain, School of Biomedical Engineering and Imaging Sciences, King’s College London, King’s Health Partners, St Thomas' Hospital, London, UK; 3 Paediatric Neurosciences, Evelina London Children’s Hospital, St Thomas’ Hospital, London, UK

**Keywords:** brain development, fMRI, neonate, preterm, somatotopic map

## Abstract

In the mature mammalian brain, the primary somatosensory and motor cortices are known to be spatially organized such that neural activity relating to specific body parts can be somatopically mapped onto an anatomical “homunculus”. This organization creates an internal body representation which is fundamental for precise motor control, spatial awareness and social interaction. Although it is unknown when this organization develops in humans, animal studies suggest that it may emerge even before the time of normal birth. We therefore characterized the somatotopic organization of the primary sensorimotor cortices using functional MRI and a set of custom-made robotic tools in 35 healthy preterm infants aged from 31 + 6 to 36 + 3 weeks postmenstrual age. Functional responses induced by somatosensory stimulation of the wrists, ankles, and mouth had a distinct spatial organization as seen in the characteristic mature homunculus map. In comparison to the ankle, activation related to wrist stimulation was significantly larger and more commonly involved additional areas including the supplementary motor area and ipsilateral sensorimotor cortex. These results are in keeping with early intrinsic determination of a somatotopic map within the primary sensorimotor cortices. This may explain why acquired brain injury in this region during the preterm period cannot be compensated for by cortical reorganization and therefore can lead to long-lasting motor and sensory impairment.

## Introduction

In the mammalian brain, the anatomical connections and neural activity of the primary sensorimotor cortices (comprising the primary motor (M1) and somatosensory cortices (S1)) are known to be functionally organized, such that information relating to a specific body part is processed in a distinct area within the contralateral cerebral hemisphere (Penfield and Boldrey 1937). Whilst there are subtle differences between M1 and S1, the general principles of this organization can be applied to both and are often pictorially represented in the classic cortical “homunculus” map; in which inferior body parts such as the feet are represented superiorly in the sensorimotor cortices (adjacent to the brain’s midline), whilst more superior body parts such as the hands and mouth are represented lower and more laterally. Body parts are also known to be disproportionately represented within the somatotopic map relative to their anatomical size, with highly innervated structures such as the mouth or fingers taking up a larger cortical area in comparison to other body regions like the trunk and legs. The resulting cortical map is thought to provide the framework for the brain’s internal body representation, thus allowing it to encode position, accurately perform motor tasks, and socially process the motor behavior and body position of others (Marshall and Meltzoff 2015).

Small animal studies suggest that a whole body topographical map emerges within the sensorimotor cortices during the equivalent period to the human late third trimester and early infancy (Seelke et al. 2012). This process is thought to be initially driven by genetic factors and feedback from spontaneously generated peripheral neural activity which activates both the primary motor and somatosensory cortices, with later experience-dependent mechanisms refining the cortical map and molding the local cortical network (Florence et al. 1996; Khazipov et al. 2004; An et al. 2014; Khazipov and Milh 2017). The critical importance of this specific period can be readily seen in studies of sensory deprivation which result in permanent alterations of S1 organization and function (Fox 1992). It is possible that this period may be similarly crucial in human infancy for the long-term development and organization of the sensorimotor cortex. This may partly explain why preterm birth (delivery less than 37 weeks gestation) engenders a significant increase in the risk of developing motor and somatosensory dysfunction, even in the absence of overt brain injury (Larroque et al. 2008; Williams et al. 2010; Setänen et al. 2016).

In recent years, Blood Oxygen Level Dependent (BOLD) contrast functional Magnetic Resonance Imaging (fMRI) has been successfully used to noninvasively characterize the cortical homunculus map of the mature human brain (Stippich et al. 1998, 1999; Moore et al. 2000; Blatow et al. 2007). These studies have confirmed that the topographical organization is highly reproducible and stable across adult populations, and have also demonstrated that fMRI has high enough sensitivity and specificity to even characterize the somatotopic map of each individual finger (Schweizer and Frahm 2009; Martuzzi et al. 2014). We have also previously shown that the combination of fMRI and custom-made robotic stimulation devices can be used to precisely map somatosensory responses in the developing sensorimotor cortex across the human preterm period (Arichi et al. 2010, 2012; Allievi et al. 2016). These studies have further highlighted the importance of this juncture for the developing sensorimotor system, as somatosensory functional responses were found to rapidly mature in preterm infants up to term equivalent age with increasing integration of activity in distinct structures such as the ipsilateral sensorimotor cortex and supplementary motor area (SMA) (Allievi et al. 2016). In addition, this maturation was found to be experience dependent with increased inter-hemispheric functional connectivity significantly correlated to greater postnatal age.

In this study, we aimed to use fMRI and a set of robotic tools for stimulating the wrists, ankles, and mouth to see whether functional responses could be somatotopically mapped in a cohort of healthy preterm infants. As our previous work has shown that functional responses in preterm infants increase their spatial specificity with maturity and occur concurrently in the primary motor and somatosensory cortices (Allievi et al. 2016), and recent evidence suggests that activity patterns in the mature sensorimotor cortex are flexibly arranged by exposure to everyday motor behavior (Ejaz et al. 2015); one possible hypothesis was that functional responses in our population would not be topographically organized. In contrast, in the context of animal studies, the alternative hypothesis was that induced responses would already be topographically organized into a cortical homunculus map even before the time of normal birth.

## Methods

The study was approved by the NHS research ethics committee and written parental consent was obtained prior to MRI/fMRI data acquisition.

### Study Population

The study population consisted of 35 preterm infants (GA at birth range: 26 + 0 to 36 + 1 weeks + days; PMA at the time of study range: 31 + 6 to 36 + 3 weeks + days) recruited from the Neonatal Intensive Care Unit (NICU) or postnatal wards of St Thomas’ Hospital, London, UK (demographic details of each infant can be found in Supplementary Table 1). All of the infants were healthy at the time of scanning and did not require any respiratory support during data acquisition. Infants were excluded from the study group if they were known to have a neurological disease or injury such as focal brain injury, a diagnosed congenital brain abnormality, and/or a clinical history of birth asphyxia or neonatal encephalopathy.

### Data Acquisition

All infants were studied during natural sleep immediately following feeding, were swaddled in a blanket and then immobilized using a vacuum evacuated bag (Med-Vac, CFI Medical Solutions, Fenton, MI, USA). Molded dental putty was placed in the external auditory meatus (President Putty, Coltene Whaledent, Mahwah, NJ, USA) and adhesive earmuffs (MiniMuffs, Natus Medical Inc., San Carlos, CA, USA) were applied in all infants to attenuate MR scanner noise. All data collection sessions were attended by a clinician (doctor or nurse) trained in neonatal resuscitation and physiological parameters (oxygen saturations, heart rate, and axillary temperature) were monitored throughout. All infants studied tolerated the study protocol well and there were no adverse events during the entire study period.

Data acquisition was performed using a 3-Tesla MRI scanner (Philips Achieva, Best, Netherlands) located on the NICU at St Thomas Hospital. BOLD contrast fMRI images were acquired with a 32 channel head coil and an EPI sequence using the following parameters: TR/TE/FA=1500 ms/45 ms/90°; resolution(x/y/z) = 2.5/2.5/3.25 mm; slice gap = 0.75 mm; non-interleaved ascending slice acquisition order; 22 slices; 256 total volumes (total time: 6 min and 34 s). For clinical reporting and image registration purposes, high resolution structural T1-weighted and T2-weighted images were also acquired for all infants studied.

**Figure 1.**
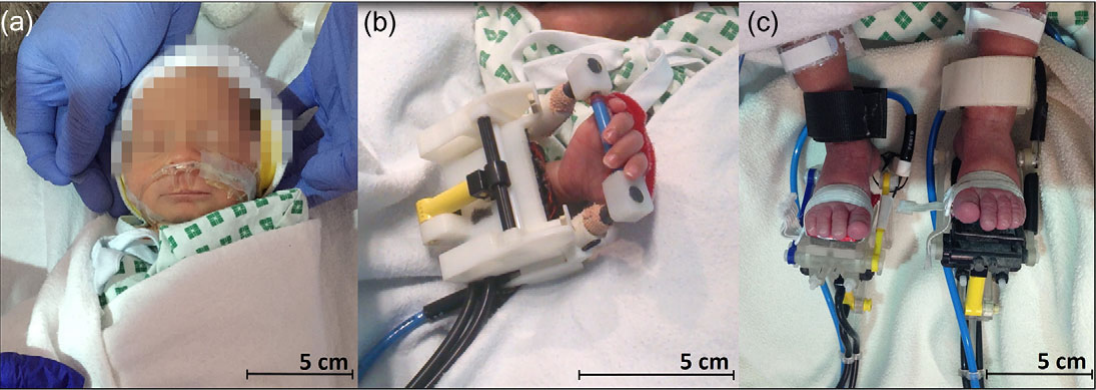
MRI-compatible automated devices used for sensory stimulation. A soft puff of air was delivered to the mouth via inverted clinical nasal cannula (*a*), while pressurized air was used to actuate the yellow piston in the robotic devices resulting in controlled flexion and extension movements of the wrist (*b*) and ankle (*c*).

A set of dedicated MR compatible robotic devices were used to induce a safe and reproducible pattern of somatosensory stimulation to different body parts across our study population (Allievi et al. 2013, 2014). These devices were custom designed and made using 3D printing to specifically fit the ankles and wrists of preterm infants (Fig. 1). Joint flexion/extension at a frequency of 0.3 Hz was achieved via a pneumatic piston driven by the hospital pressurized air supply, which was computer-controlled from the MR scanner control room and synchronized with image acquisition. For detailed description of the wrist and ankle robotic devices please refer to Arichi et al. (2010), Allievi et al. (2013). To provide a somatosensory stimulus to the mouth, we repurposed clinical nasal cannulae which are usually used to provide supplemental oxygen therapy. Nasal cannulae were chosen so as to minimize the amount of equipment attached to the infant’s face (and thus prevent discomfort) and to ensure that clinical infection control measures were followed with single-use equipment. The cannulae were fit around the baby’s head but with the prongs orientated downwards so that a gentle puff of air (0.4 atm at 0.3 Hz) could be delivered to the area between the nose and lips. The pattern of stimulation, timing and amplitude of all patterns of stimulation were constantly monitored on a user interface displayed on a PC connected to the control box (Labview, National Instruments, Austin, TX, USA). Every experiment consisted of an identical “on-off” block paradigm in which a single stimulus type was presented (i.e., only one joint was stimulated at a time across the entire run). Each of the 5 experiments (stimulating either the left or right wrist, the left or right ankle, or mouth) consisted of a total of 8 blocks containing 24 s of stimulation interleaved with 24 s of rest. A given infant in the study population was involved in a maximum of 4 experiments with a different type of stimulation, which were chosen at random prior to data collection inside the MRI scanner (full details of each infant can be seen in Supplementary Table 1).

### Data Analysis

fMRI data analysis was performed using tools implemented in FMRIB’s Software Library (FSL, www.fmrib.ox.ac.uk/fsl) (Smith et al. 2004).The raw images were first visually assessed for evidence of severe image artifacts or a large amount of head motion which would not be amenable to correction and data was discarded accordingly. Head motion was quantified from displacement parameters derived from rigid body head realignment to the reference (center) volume and the calculation of the Root Mean Square (RMS) intensity difference of volume N to the reference. Corrupted data points were dealt with by deleting contiguous blocks of data if the absolute displacement during a volume exceeded 1.25 mm (equal to half the in-plane resolution); and by using the RMS intensity difference metric to define a binary confound regressor for the later general linear model (GLM) analysis (akin to “motion scrubbing” (Power et al. 2012)). Raw data were then pre-processed using an optimized pipeline for neonatal subjects implemented in FEAT (FSL’s Expert Analysis Tool v5.98), consisting of slice time correction, high pass filtering (cut-off 50 s), non-brain tissue removal using BET (brain extraction tool), global intensity normalization and spatial smoothing (Gaussian filter of 5 mm FWHM) (Arichi et al. 2010). Additional data denoising was performed using independent component analysis (ICA) to remove signal artifacts related to the non-linear effects of head motion and physiological effects such as cardiovascular pulsation and respiratory movements (Beckmann et al. 2005). A univariate (voxel-wise) analysis was then performed using the GLM, with the stimulation paradigm convolved with an optimized set of basis functions derived from an age-specific hemodynamic response function (HRF) (Arichi et al. 2012). To further deal with the possible confounding effects of head motion, additional confound regressors were also included in the GLM analysis including extended head motion parameters (head translation and rotation, their squares, the derivatives, and the square of the derivatives) and the binary regressors derived from the first stage of pre-processing. The resulting t-statistical images were converted to a normally distributed z-statistical image and a threshold of 2.3 (with a corrected cluster significance level of *P* < 0.05) was defined to generate individual subject activation maps.

Lower level functional activation maps were then registered to the subject’s own high resolution T2-image using rigid-body registration and then to an age-specific template brain (Serag et al. 2012) using a non-linear registration. Group analysis (controlling for gestational age at birth and PMA at scan) was then performed separately for each of the body areas stimulated using a nonparametric one-sample *t*-test implemented with permutation methods and threshold free cluster enhancement (TFCE) (family wise error (FWE) corrected *P* < 0.05) using Randomise (v2.0) (Nichols and Hayasaka 2003; Smith and Nichols 2009). For the final characterization of the somatotopic map of functional responses, only the main cluster within the sensorimotor cortices was considered, therefore additional areas of activation such as within the insulae following mouth stimulation were not included. Group response maps were thresholded (*P* = 0.05) and combined together using a “winner-takes-all” approach, so that voxels containing overlapping functional responses were assigned to the dominant cluster. The final results of these group analyses were then projected for visualization onto the inflated cortical surface of an age-appropriate template brain using MIRTK (mirtk.github.io).

## Results

Data was successfully collected in 49/68 experimental sessions following discard of data corrupted by excessive head motion or image artifact. Successful data was collected in 10 of 17 subjects with left ankle somatosensory stimulation (median PMA: 35 + 2 weeks; range: 33 + 6–36 + 3 weeks); 9 of 14 subjects with right ankle stimulation (median PMA: 34 + 4 weeks; range: 31 + 6–36 + 3 weeks); 10 of 12 subjects with left wrist stimulation (mean PMA: 34 + 2 weeks; range: 33 + 0–35 + 3 weeks); 10 of 12 subjects with right wrist stimulation (median PMA: 34 + 2 weeks; range: 33 + 3–36 + 1 weeks); and 10 of 13 subjects who received stimulation of the mouth (median PMA: 35 + 1 weeks; range: 32 + 3 to 36 + 3 weeks) (demographic information of the included study population is reported in Table 1). All of the infants were reported as having appropriate brain appearances on their structural images. Four of the infants had unilateral grade 1 intraventricular hemorrhage (IVH) and 3 of the infants had a small number of punctate white matter lesions (see Supplementary Table 1).

**Table 1:** Demographic information of the final study population for each stimulus type

Body part group	*n*	GA at birth in weeks median (range)	Birth weight in grams median (range)	PMA at scan in weeks median (range)
Left ankle	10	34 + 2 (28 + 3–36 + 1)	1850 (1120–3110)	35 + 2 (33 + 6–36 + 3)
Left wrist	10	32 + 3 (26 + 3–34 + 5)	1930 (840–2330)	34 + 2 (33 + 0–35 + 3)
Right ankle	9	33 + 3 (28 + 5–35 + 4)	2100 (1340–3110)	34 + 4 (31 + 6–36 + 3)
Right wrist	10	32 + 4 (29 + 1–35 + 6)	1680 (1330–2100)	34 + 2 (33 + 3–36 + 1)
Mouth	10	34 + 1 (30 + 4–35 + 4)	1980 (1440–3110)	35 + 1 (32 + 3–36 + 3)

GA, gestational age at birth in weeks; PMA, postmenstrual age at scan in weeks.

**Figure 2.**
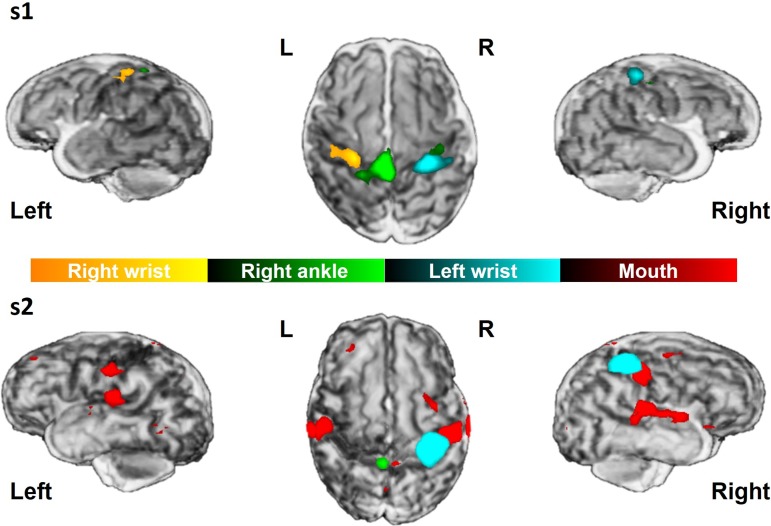
Representative functional responses in two subjects scanned at 33 + 6 weeks PMA (s1) and 35 + 3 weeks (s2). Single subject results show distinct significant clusters of functional activation (thresholded at *z* = 2.3) following stimulation of different body parts overlaid on the subject’s own 3D rendered T2-weigthed image.

In all subjects, passive movement of a single joint resulted in a significant cluster of positive BOLD response in a localized area within the sensorimotor cortex spanning both S1 and M1 across the central sulcus contralateral to the body part stimulated (Fig. 2). As seen in the mature somatotopic “homunculus” map, clusters of functional activation relating to ankle movement were located superiorly to those of the wrist which were located on the superior medial portion of the central sulcus “hand-knob” (Yousry et al. 1997; Hlustik et al. 2001). In addition, wrist stimulation induced significantly larger clusters of functional activation (median volume: 2737.33 mm^3^; range: 478.54–11 306.65 mm^3^) in comparison to those following ankle stimulation (median volume: 1208.41 mm^3^; range: 252.6–17 209.083 mm^3^) (Wilcoxon rank sum test, *P* = 0.0151). Somatosensory stimulation of the mouth induced a bilateral pattern of functional activity which was situated inferiorly and laterally to the wrist response within the sensorimotor cortex.

**Figure 3.**
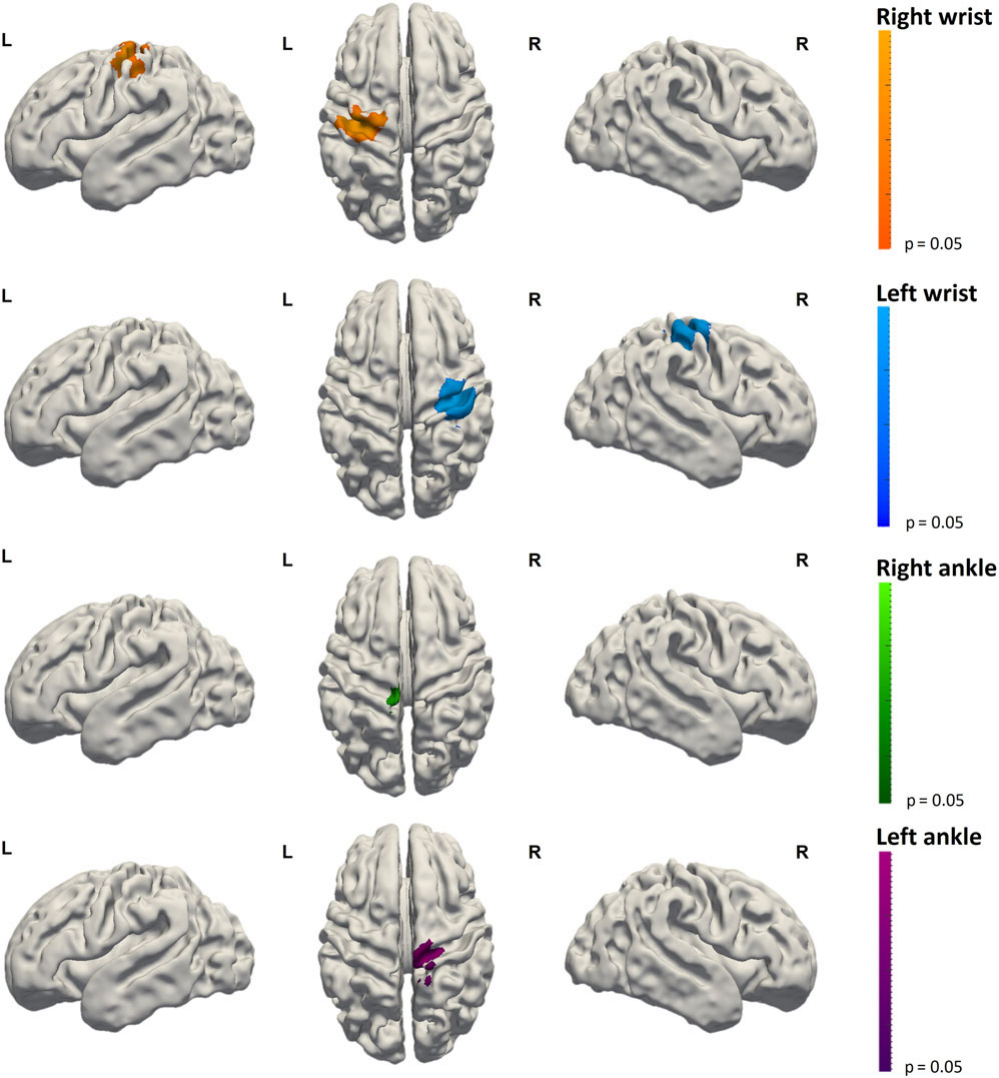
Functional responses resulting from the group analysis following somatosensory stimulation of the left ankle (*n* = 10), left wrist (*n* = 10), right wrist (*n* = 10), and right ankle (*n* = 9). Well localized distinct clusters of activation can be seen within the contralateral sensorimotor cortex across the central sulcus. Images show the results of one-sample nonparametric *t*-tests (*P* < 0.05 corrected for family wise error) projected onto the gray-white matter boundary of a 34 week PMA template brain.

**Figure 4.**
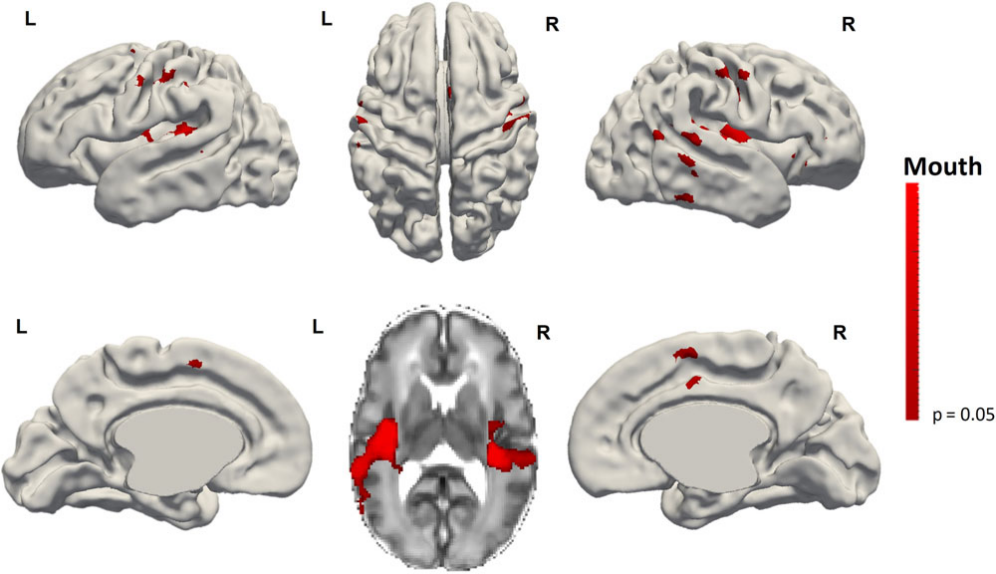
Result of the group analysis of functional responses following mouth stimulation (*n* = 10). Clusters of activation can be seen within the bilateral sensorimotor cortices. Additional clusters of activation were also seen in the midline Supplementary Motor Area (SMA) (lower row left and right figures) and bilaterally within the insulae (lower row, center image). Images show the results of one-sample nonparametric *t*-test (*P* < 0.05 corrected for family wise error) projected onto the gray-white matter boundary of a 34 week PMA template brain.

Activation following wrist stimulation was also seen in some infants within the ipsilateral sensorimotor cortex (8/20, 5 subjects in the right wrist group and 3 in the left wrist group) and supplementary motor area (SMA) (10/20, half of the subjects in each group). In contrast, SMA activation following ankle stimulation was seen in less subjects (5/19) compared to wrist. Clusters of identified activity for a given side of stimulation (i.e., the right wrist) appeared to be symmetrical with those seen following stimulation of the opposite side (i.e., the left wrist). Of interest, when clusters of ipsilateral activity were seen, they were located in an overlapping region to that of the primary response cluster to stimulation of the same limb on the opposite side, suggesting that this activity was occurring in its functional homolog within the ipsilateral hemisphere. However at a group level, functional clusters in the ipsilateral hemisphere and supplementary motor area did not reach significance for any of the individual limb stimulation groups (Fig. 3). Mouth stimulation was also associated with additional clusters of activity in the insular cortices and SMA (Fig. 4).

**Figure 5.**
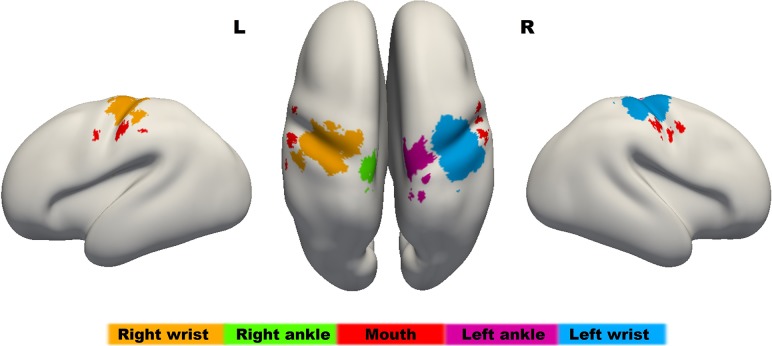
The sensorimotor homunculus in the preterm human brain at 34 weeks PMA. The map has been overlaid onto an age-specific inflated brain template using a “winner-takes-all” approach after combing the significant results of the group level activation maps from each stimulated body part. In agreement with the well characterized adult somatotopic map, functional activity relating to the ankles (green and purple) is located superiorly to those of the wrist (orange and blue) and mouth (red). This map will be made publically available for download from brain-development.org.

Distinct localization of functional responses following stimulation of different body parts into a somatotopic representation could be clearly appreciated when combined into a single “homunculus” map (Fig. 5). As has been characteristically described in the adult topography, clusters of activation corresponding to the somatosensory stimulation of the ankle were identified superiorly and adjacent to the midline within the sensorimotor cortices; clusters corresponding to stimulation of the wrist were located infero-laterally to those of the ankle; and clusters relating to mouth stimulation were located inferior and lateral to those of the wrist.

## Discussion

Using fMRI and specific patterns of precisely controlled somatosensory stimulation, we have been able to carry out the most detailed characterization to date of cortical somatotopy in the preterm human brain. Our results demonstrate that there is a clear correspondence between sensory information related to distinct body parts and specific areas within the developing sensorimotor cortex even before term equivalent age. The topography of the identified cortical representation closely resembles that of the well described “homunculus” map of the mature brain, with inferior body parts mapping to the superior cortex and highly innervated body regions disproportionately mapping to larger area of cortex relative to their physical size (Penfield and Boldrey 1937).

Functional specialization has long been recognized as a hallmark feature of the brain ever since it was first identified that the cortex could be histologically parcellated on the basis of its cytoarchitectonic features (Brodmann 1909). Within this framework, function within a given cortical region is tightly constrained by its anatomical microstructure and the underlying pattern of its structural connections (Passingham et al. 2002). Imaging methods have since made it possible to confirm the specific roles of the primary sensory cortices and have enabled precise mapping of their receptive fields thus enabling a new understanding of their functional organization (Blankenburg et al. 2003; Wandell et al. 2007). This has included several studies which have characterized a topographical map in both S1 and M1 which is largely in agreement with Penfield’s classical homunculus, including its two areas of major discontinuity (between the hands and the feet in both S1 and M1; and the feet and the genitalia in S1 only) (Nakamura et al. 1998; Kocak et al. 2009; Heed and Röder 2010; Parpia 2011). This kind of topographical organization is thought to have evolved to provide an optimal substrate for efficient neural processing within the geometric, biophysical, and energy constraints of the brain (Laughlin and Sejnowski 2003) and facilitates social interaction by enabling registration of correspondence in body position and behavior between self and others (Marshall and Meltzoff 2015).

Whilst a relatively precise correspondence between functional and architectonic parcellation of areas such as S1 can be readily seen in a mature brain, the factors which underlie its ontogeny remains unclear (Parpia 2011). One possibility is that of a cortical “protomap” whereby the location and function of neurons are controlled initially by genetic factors which mediate spatially specific molecular signaling within neural progenitor cells (Rakic 1988). In contrast, it has also been suggested that neuronal function within an initially homogenous cortex is defined by afferent thalamic inputs and activity-dependent mechanisms through early environmental influences (Sur and Rubenstein 2005). Our results and those of developmental animal studies suggest that both factors contribute together at different but overlapping times, as architectonic maps (including a putative barrel cortex) appear to emerge within S1 as early as the embryonic stage, whilst topographic functional maps do not develop until much later in postnatal life (Seelke et al. 2012). Distinct but not topographically organized body part representation can be seen in the S1 of rats as early as P5-10, before more precise organization emerges during the subsequent period leading up to P15, and an adult-like pattern is eventually established by P20 (Seelke et al. 2012). This initial mismatch between cytoarchitecture and function therefore supports a switch from genetically driven mechanisms to a subsequent activity driven cortical refinement process which is influenced by the establishment of thalamic connectivity as suggested by the radial-unit hypothesis (Rakic 1988). In agreement with this, very early genetic alteration during gestation yields an aberrant architectonic map (Fukuchi-Shimogori and Grove 2001; Ragsdale and Grove 2001), whilst later abnormal afferent information significantly alters functional maps but not the anatomical location of S1 (Fox 1992; Feldman and Brecht 2005).

In the last trimester of human gestation ascending thalamo-cortical axonal pathways and cortico-cortical axons grow through the transient subplate layer and establish the cortex’s lifelong framework of connectivity (Florence et al. 1996; Pallas 2001; López-Bendito and Molnár 2003). By the latter stages of the preterm period (>33 weeks PMA), the subplate decreases in thickness particularly in the parietal lobes (more so than in the temporal or frontal lobes) as these longer afferent pathways connect into the cortical plate allowing activity-dependent elaboration and refinement of the initial topographical map (López-Bendito and Molnár 2003; Kostović and Jovanov-Milošević 2006; Perkins et al. 2008). Therefore whilst the underlying cytoarchitecture and functional role of the sensorimotor cortices is already established, ex-utero experience during the preterm period could potentially influence the further development of precise cortical topographical maps (Allievi et al. 2016). This may explain in part why preterm birth and specifically perinatal sensorimotor network injury markedly increases the risk of developing conditions such as cerebral palsy which are associated with long-term sensory and motor impairment (Fawke 2007; Larroque et al. 2008; Williams et al. 2010; Arichi et al. 2014). Whilst previous work has found that afferent thalamo-cortical pathways can grow around areas of brain injury acquired in the preterm period (Staudt et al. 2004; Arichi et al. 2014), our results provide essential neonatal validation of studies in older children and young adults which have found that perinatal damage to the primary somatosensory cortex cannot be compensated for through neuroplasticity or cerebral reorganization (Juenger et al. 2011).

In keeping with previous studies in both preterm infants and equivalent animal models, we saw that peripheral somatosensory stimulation induced clear patterns of functional activity across the contralateral peri-rolandic region encompassing both M1 and S1 (Khazipov et al. 2004; An et al. 2014; Allievi et al. 2016). This seemingly concurrent activity is thought to occur through numerous direct cortico-cortical connections between M1 and S1 (Farkas et al. 1999; Ghosh et al. 2010; An et al. 2014). During early life, this connectivity pathway is of particular importance as sensory feedback from peripherally generated spontaneous limb movements are thought to play a crucial role in the early development and refinement of the immature motor cortex (Khazipov et al. 2004; Yang et al. 2009; An et al. 2014). Additional functional activity in the ipsilateral sensorimotor cortex and SMA were also seen in a subset of patients, predominately following stimulation of the wrist and mouth. This is in agreement with our previous work which found a wider pattern of functional response with increasing age (Wang et al. 2007; Allievi et al. 2016). Our finding that ankle stimulation responses occur predominately in the contralateral sensorimotor cortex and without involvement of the SMA suggest that maturation of this dispersed network response may occur with different trajectories for distinct body parts, perhaps corresponding to different levels of sensory experience or an intrinsic mechanism which predisposes these regions to allow complex motor behavior such as sucking or grasping soon after birth.

In addition to localization of functional responses following limb stimulation, we were also able to identify further inferior and lateral clusters of bilateral functional activity within the primary sensorimotor cortices and insulae, as well as the SMA following somatosensory stimulation of the mouth. These findings are in keeping with adult fMRI studies which have demonstrated that the human oral area is densely innervated with a wide network of functional connections to other distinct areas across the cortex (Stippich et al. 1999; Miyamoto et al. 2005). The insula is involved in the elaboration of a wide variety of sensory processes (Penfield and Faulk 1955) including pain, thermal coding, gustatory sensation, and intraoral somatosensory processing (Zald and Pardo 2000). Whilst there were differences in our study with respect to the type of stimulus presented to the mouth (predominately tactile) and limbs (both tactile and proprioceptive), this distinction is likely to be of less significance as both types of stimuli are communicated within a final common thalamo-cortical pathway to the primary sensorimotor cortex where processing is not modality-specific during the preterm period (Fabrizi et al. 2011). SMA activation may also be partly explained by the essential role of tactile sensation in and around the mouth in early human life to elicit sucking activity.

Taken together, our results suggest that in the late preterm period, maturing patterns of connectivity acting on a genetically determined sensorimotor “protomap” are shaping the size of the pre-determined somatotopic functional areas and establishing their wider patterns of network activity. It will therefore be crucial to next study how this connectivity is maturing in both a functional and structural sense using measures such as those derived from other complementary methods such as diffusion MRI. With this in mind, we will make the homunculus map publically available for download (http://brain-development.org/). It is also important to consider that we cannot definitively extrapolate our findings to the fetal sensorimotor system development and therefore it will be vital to study how this putative homunculus topographic map compares to that of infants delivered at full term gestation, how it evolves throughout later infancy and how it may be altered by specific patterns of brain injury.

## Conclusion

In the human preterm period, functional activity within the sensorimotor cortices is already somatotopically organized in a pattern similar to the classic mature “homunculus” representation. This result suggests that as described in animal models, the establishment of this organization is first driven by genetic factors ready for later elaboration through experience-driven changes in connectivity. Given that preterm infants are constantly exposed to entirely different sensory experiences in the ex-utero environment, our findings further emphasize that the human preterm period may represent a critical window of vulnerability for altered sensorimotor cortex development, which may explain the high incidence of functional motor and sensory difficulties in this population.

## Supplementary Material

Supplementary Data
