# Supplementary material for: Somatotopic Mapping of the Developing Sensorimotor Cortex in the Preterm Human Brain
Source: Cereb Cortex. 2018 Apr 18;28(7):2507–15. doi: 10.1093/cercor/bhy050 (PMC5998947; doi:10.1093/cercor/bhy050)
Supplement: Supplementary Data [file supplementary_information.reviewed.docx]

| **Subject** | **GA** | **PMA** | **HC** | **W (g)** | **Stimuli received** | **Clinical MRI appearance** |
| --- | --- | --- | --- | --- | --- | --- |
| 1 | 34+0 | 35+4 | 28.7 | 1550 | RW | normal |
| 2 | 31+3 | 33+3 | 31.1 | 1410 | RW | normal |
| 3 | 29+1 | 34+1 | 30 | 1570 | RW | grade 1 IVH |
| 4 | 33+3 | 34+4 | 29.5 | 1960 | RA,RW | 6 punctate lesions |
| 5 | 32+0 | 33+0 | 30.0 | 1930 | RA,RW | normal |
| 6 | 28+5 | 31+6 | 26.0 | 1280 | RA | normal |
| 7 | 32+2 | 34+1 | 31.0 | 2010 | RA,RW | grade 1 IVH |
| 8 | 34+1 | 35+4 | 32.0 | 1950 | LA | normal |
| 9 | 26+0 | 33+6 | 31.0 | 1580 | LW | normal |
| 10 | 32+6 | 33+3 | 32.0 | 1870 | RW | normal |
| 11 | 31+4 | 34+1 | 29.2 | 1670 | RW | normal |
| 12 | 32+2 | 34+4 | 28.0 | 1645 | RW | normal |
| 13 | 35+3 | 36+1 | 31.0 | 1890 | RW | 2 punctate lesions & grade 1IVH |
| 14 | 35+6 | 36+1 | 30.5 | 1700 | RW | normal |
| 15 | 33+4 | 34+3 | 29.5 | 1684 | RW | normal |
| 16 | 36+1 | 36+3 | 30.0 | 2380 | LA,LW | normal |
| 17 | 28+3 | 34+4 | 30.5 | 2260 | LA,RW,LW | normal |
| 18 | 28+3 | 34+4 | 30.0 | 1840 | LW | Grade 1 IVH, mild lateral ventricular asymmetry |
| 19 | 32+4 | 33+6 | 29.4 | 1690 | RA,RW,LW | normal |
| 20 | 32+4 | 33+6 | 28.0 | 1590 | LA,LW | normal |
| 21 | 33+2 | 34+5 | 31.5 | 2250 | LA,LW | normal |
| 22 | 34+4 | 35+0 | 31.0 | 2000 | LA,RA,RW | normal |
| 23 | 34+5 | 35+3 | 30.0 | 1840 | LA,RA,LW,M | normal |
| 24 | 32+5 | 33+6 | 30.0 | 1710 | LA,RA,LW,M | normal |
| 25 | 35+1 | 35+4 | 30.0 | 2170 | LA,RA,M | normal |
| 26 | 34+3 | 35+1 | 29.5 | 1600 | LA,RA,M | normal |
| 27 | 34+3 | 35+2 | 29.0 | 1500 | LA,RA,M | normal |
| 28 | 35+4 | 36+3 | 35.0 | 2715 | LA,RA,M | normal |
| 29 | 30+4 | 32+3 | 28.0 | 1345 | LA,RA,M | 5 punctate lesions |
| 30 | 35+3 | 35+5 | 32.5 | 2680 | LA,RA,M | normal |
| 31 | 35+1 | 35+3 | 34.0 | 2550 | LA,M | normal |
| 32 | 33+2 | 33+6 | 31.7 | 1910 | LA,M | normal |
| 33 | 33+4 | 35+1 | 32.0 | 2400 | M | normal |
| 34 | 33+1 | 34+4 | 28.0 | 1450 | LA,M | normal |
| 35 | 33+6 | 34+2 | 31.0 | 1860 | M | normal |

***Supplementary table 1: Demographic information of the recruited study population and the stimulus type received.*** *GA= gestational age at birth in weeks + days; PMA= Post Menstrual Age at scan in weeks + days; HC= Head circumference at scan in cm; W= Weight at scan in grams; RW= right wrist, RA= right ankle, LA= left ankle; LW= left wrist; M=mouth; IVH= intraventricular haemorrhage*
